# Towards Scalable Large-Area Pulsed Laser Deposition

**DOI:** 10.3390/ma14174854

**Published:** 2021-08-26

**Authors:** Zakhar Vakulov, Daniil Khakhulin, Evgeny Zamburg, Alexander Mikhaylichenko, Vladimir A. Smirnov, Roman Tominov, Viktor S. Klimin, Oleg A. Ageev

**Affiliations:** 1 Federal Research Centre, Southern Scientific Centre of the Russian Academy of Sciences (SSC RAS), 41 Chekhov St., 344006 Rostov-on-Don, Russia; 2 Research Laboratory of Functional Nanomaterials Technology, Southern Federal University, 2 Shevchenko St., 347922 Taganrog, Russia; dhahulin@sfedu.ru; 3 Department of Electrical & Computer Engineering, National University of Singapore, 4 Engineering Drive 3, Singapore 117583, Singapore; zamburg@nus.edu.sg; 4 FORS Development Center, 3, Trifonovskiy Tupik, 129272 Moscow, Russia; alexandrVM@bk.ru; 5 Institute of Nanotechnologies, Electronics and Equipment Engineering, Southern Federal University, 2 Shevchenko St., 347922 Taganrog, Russia; vasmirnov@sfedu.ru (V.A.S.); tominov@sfedu.ru (R.T.); kliminvs@sfedu.ru (V.S.K.); ageev@sfedu.ru (O.A.A.); 6 Research and Education Centre ‘Nanotechnologies’, Southern Federal University, 2 Shevchenko St., 347922 Taganrog, Russia

**Keywords:** pulsed laser deposition, nanomaterials, computer simulation, thin films, large-area deposition, metal oxides, thickness uniformity, ablation, lithium niobate

## Abstract

One of the significant limitations of the pulsed laser deposition method in the mass-production-technologies of micro- and nanoelectronic and molecular device electronic fabrication is the issue of ensuring deposition of films with uniform thickness on substrates with large diameter (more than 100 mm) since the area of the laser spot (1–5 mm^2^) on the surface of the ablated target is incommensurably smaller than the substrate area. This paper reports the methodology that allows to calculate the distribution profile of the film thickness over the surface substrate with a large diameter, taking into account the construction and technological parameters of the pulsed laser deposition equipment. Experimental verification of the proposed methodology showed that the discrepancy with the experiment does not exceed 8%. The modeling of various technological parameters influence on the thickness uniformity has been carried out. Based on the modeling results, recommendations and parameters are proposed for manufacturing uniform thickness films. The results allow for increasing the film thickness uniformity with the thickness distribution < 5% accounts for ~ 31% of 300 mm diameter substrate.

## 1. Introduction

Currently, the pulsed laser deposition (PLD) method is widely used to form epitaxial and single-crystal complex oxides films with ferroelectric, ferromagnetic, dielectric, and superconducting properties [1,2,3,4,5]. The advantages of this method include the possibility of maintaining the stoichiometric composition of the ablated material [6], good film adhesion [7], versatility in choosing the deposited material, as well as the possibility of forming film coatings on the surface of thermosensitive materials [8]. These advantages allow to use of the PLD method in the formation of energy harvesting devices [9], optical structures [10], sensor elements [11], ferroelectric films [12], memristor structures [13,14], and medical coatings [15]. Thus, the study of the PLD process and the development of methods for solving issues limiting its implementation in mass production is highly urgent.

In the PLD method, a laser beam is focused on a target placed in a vacuum chamber. The target material is ablated under the influence of laser radiation of high-power density, as the result the ablated particles are deposited on the substrate. Although the PLD method is widely used in scientific research [16,17,18,19,20,21,22], its industrial application is limited [1,23]. One of the significant issues of PLD in mass-production-technologies of micro- and nanoelectronic device fabrication is ensuring uniform thickness films deposition on large diameter substrates (more than 100 mm), since the area of the laser spot (1–5 mm^2^) on the surface of the ablated target is incommensurably smaller than the substrate area [24].

In the modern micro- and nanoelectronic industry, the standard is the non-uniformity of the film thickness over the surface of the entire substrate ~5%, except for 5 mm at the edge. Since the PLD method is relatively new [25,26] and its integration with industrial technologies of micro- and nanoelectronics is still at its initial stages. The study of regularities and the search for methods to achieve the indicated values of non-uniformity on substrates of large diameter (more than 100 mm) requires additional research. In [27,28,29,30,31,32,33,34], several approaches for forming films with a uniformity of 70–95% on substrates with a diameter of 100–200 mm are described in detail. However, obtaining uniform of films by PLD on 300 mm-diameter substrates is still challenging and often requires using scanning systems that ensure the movement of the laser beam over the target surface and the movement of the substrate [1]. One of the possible ways to overcome this limitation is to use a laser beam scanning system to move the laser beam over the target surface, rotate the target and substrate, and optimize the parameters of these processes, considering the geometry of the growth chamber.

The purpose of this work is to study the processes affecting film thickness uniformity deposited by the PLD method on substrates of large diameter (100 mm and more) and developing the methodology that allows calculating the film thickness distribution profile over the substrate surface, taking into account the design and technological parameters of PLD equipment. The results presented in this work are partially based on the previous studies, conducted both in our laboratory as well as by other researchers [27,28,29,30,31,32,33,34]. We implemented a new approach to the calculation of a trajectory of a laser beam along the target surface (Video S1 and S2). These improvements made it possible to describe a trajectory of a laser beam along the target surface much more correctly and to take into account the influence of a larger number of scanning parameters. As a result, we obtained quantitatively more accurate results of the film thickness spatial distribution.

## 2. Materials and Methods

Modeling the PLD process, assessing technological parameters and their influence on the film thickness uniformity were carried out in the MATLAB software (MathWorks Inc., Natick, MA, USA). The process of scanning a target with a diameter of up to 50 mm by a laser beam is considered on the example of cluster nanotechnological complex NANOFAB NTK-9 (NT-MDT, Zelenograd, Russia), comprising of a Pioneer 180 PLD module (Neocera LCC, Beltsville, MD, USA). The parameters used in the modeling are presented in Table 1. The studied parameter took the values indicated in the table, while all the others remained unchanged (highlighted in Table 1), to estimate the influence of one of the scanning system parameters on the films thickness uniformity.

**Figure 1:**
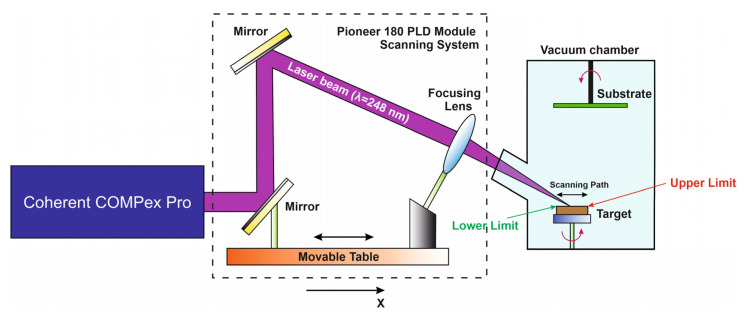
Laser beam scanning of the target surface in the Pioneer 180 PLD module.

The movement of the laser beam over the target surface is carried out using a scanning system consisting of a pair of mirrors and a focusing lens fixed on a movable stage. The laser beam hits the mirrors and re-reflected on the focusing lens. Then the laser beam hits the target surface through the window in the growth chamber. The movable stage is part of the module’s scanning system, moves along the *x*-axis, thereby scanning the laser beam along the target surface (Figure 1).

The *Upper Limit* and *Lower Limit* parameters are the lower and upper limits of the laser beam movement and allow localizing the area of action of the laser beam on the target surface by a segment (*Lower Limit*, *Upper Limit*). The point in this segment at which the irradiation process starts is determined by the *Origin* parameter. Values of all of the aforementioned parameters lie within [0, 2*R*], where *R*—target radius. The *Max Velocity* and *Min Velocity* parameters determine the maximum and minimum speed of movement of the laser beam on the target surface. Near the point defined by the *Origin* parameter, the scanning speed varies within (*Min Velocity*, *Max Velocity*). On the (*Origin*, *Upper Limit*) section, the change in scanning speed is determined by the *Upper Coefficient* parameter, and on the (*Lower Limit*, *Origin*) section by the *Lower Coefficient* parameter.

The speed of scanning system movement is described by two functions that intersect at a point with a coordinate defined by the *Origin* parameter. The relationship between the functions is described by the expression:

(1) $$V_{a}(x)=\frac{V_{\max}\cdot R_{u}}{C_{\upsilon }(x-Origin)},$$

where $V_{\max}$—maximum speed of the scanning system, $R_{u}$ is defined as the largest of two values |*Lower Limit–Origin*| and |*Upper Limit–Origin*|, and the coefficient $C_{\upsilon }$ is equal to *Lower Coefficient* for all *x < Origin* and *Upper Coefficient* for all *x > Origin*.

The mutual arrangement of the centers of the target and the substrate is not coaxial; therefore, to form a continuous film, the substrate rotates with a certain angular velocity, since the scanning system ensures the movement of the laser beam only along the diameter of the target. The target also rotates with an angular velocity to ensure uniform irradiation. The motion control of the scanning system is carried out using the Pioneer 180 PLD software (Neocera LCC, Beltsville, MD, USA).

It is necessary to describe the spatial distribution of molecules in directions during target ablation to estimate the uniformity of film deposition on a substrate. Since the laser spot has a small area, and the crater on the target surface after exposure to the laser pulse has a depth of about several nanometers [35,36], the volume of the material removed in one pulse is also relatively small. Thus, the area of laser action can be considered as a Knudsen cell, consisting of an isothermal shell with an infinitely small hole $dA_{e}$ and infinitely thin walls (supplementary materials Figure S1) [37].

The ablation region contains *N* atoms that collide with the cell walls and are reflected from them without changing the velocity. Atoms moving towards the hole leave the cell at the same speed. The expression determines the distribution of the velocities of atoms in the flow of matter for a small number of atoms within a small spatial angle $d\omega_{s}$. The angle φ determines the direction of movement with respect to the normal of the hole $dA_{e}$. It is possible to obtain the mass of the substance deposited per unit area taking into account the distribution of the velocities of atoms in directions [36]:

(2) $$\frac{dM_{r}(\varphi ,\theta )}{dA_{r}}=\frac{M_{e}}{\pi r^{2}}\cos\varphi \cos\theta ,$$

where $M_{e}$—mass of vaporized matter.

From Equation (2), it follows that the propagation of matter occurs mainly in directions close to the normal to the evaporated surface (cosφ → max). Equation (2) can be simplified, since in the PLD module, the substrate is located parallel to the target, the angles *φ* and *θ* are equal, and the cosines of these angles are equal to *h/l*, and the distribution of the film thickness on the substrate is described by the expression [36]:

(3) $$d=\frac{M_{e}}{\pi \rho_{u}h^{2}{\left[1+{\left(l/h\right)}^{2}\right]}^{2}},$$

where *l*—the current coordinate along the substrate radius, *h*—target-substrate distance [38], mass of ablated substance $M_{e}=\rho_{u}V_{a}$; $V_{a}=S_{las.spot}\cdot \Delta z$, Δz—target material ablation depth, $S_{las.spot}$—laser spot area, and $\rho_{u}$—target material density.

It is convenient to characterize the uniformity of film deposition over the diameter of the substrate using the ratio:

(4) $$\frac{d}{d_{\max}}=\frac{d(l)}{d(0)}={\left[1+{\left(l/h\right)}^{2}\right]}^{-2},$$

where $d_{\max}$—maximum film thickness. The value of the relative thickness allows one to compare films obtained at different values of technological parameters, regardless of the absolute values of the film thickness.

We introduce a parameter $\Omega_{95}$ that is defined as:

(5) $$\Omega_{95}=\frac{S_{95}}{S},$$

where *S*_95—_the substrate area where relative thickness lies within [0.95, 1] and *S*—area of the entire substrate. If the relative thickness allows one to conclude the uniformity of the film, then the value of the $\Omega_{95}$ parameter makes it possible to characterize the distribution of the relative film thickness from the point of view of industrial application (Figure S2). In particular, using the $\Omega_{95}$ parameter, it is possible to estimate the size of the substrate for the deposition of the films with thickness uniformity is 5% by the PLD method under specific scanning modes.

The methodology considered above does not take into account the size of the laser spot on the target surface and the associated with “flip-over effect” [37]. In [39], the equation describes the thickness of a film on a substrate, taking into account the gas dynamics of three-dimensional vapor expansion during PLD and the associated evolution of the shape of the laser plume was proposed:

(6) $$d=d_{\max}{\left(1+\frac{1}{p}tg^{2}\theta_{x}+\frac{q^{2}}{p}tg^{2}\theta_{y}\right)}^{-3/2},$$

where *p* and *q*—coefficients are obtained from the solution of the system of gas-dynamic equations and $\theta_{x}$ and $\theta_{y}$—the corresponding components of the particle scattering angle. However, solving the system of equations for finding the coefficients *p* and *q* significantly complicates the calculations.

The film growth process during PLD depends on many different factors [40,41,42,43,44,45,46]. To simplify the methodology, the following assumptions have been made: laser ablation occurs in a vacuum; the target surface is considered ideally smooth under the PLD process (there is no laser modification of the target surface); there is no desorption of the deposited material from the substrate surface; the shape of the laser plume in the process of moving towards the substrate remains unchanged.

**Figure 2:**
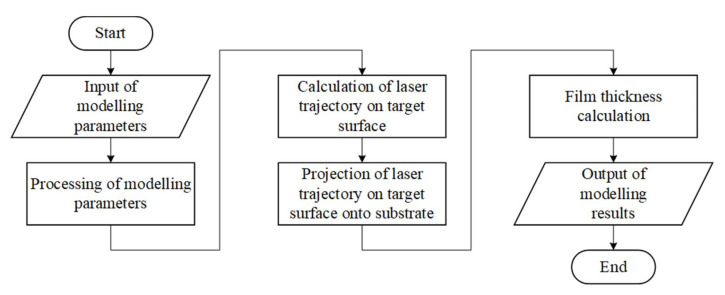
The main stages of films thickness calculation.

Figure 2 shows the calculation of the films thickness algorithm based on the developed methodology (1)–(6).

In the block for input of modeling parameters, technological parameters that determine the modes of scanning the laser beam over the target surface, as well as the rotation speed of the target and substrate are set.

In the block for processing of modeling parameters, a check for the consistency of the given parameters and their transformation to a single frame of reference is made.

**Figure 3:**
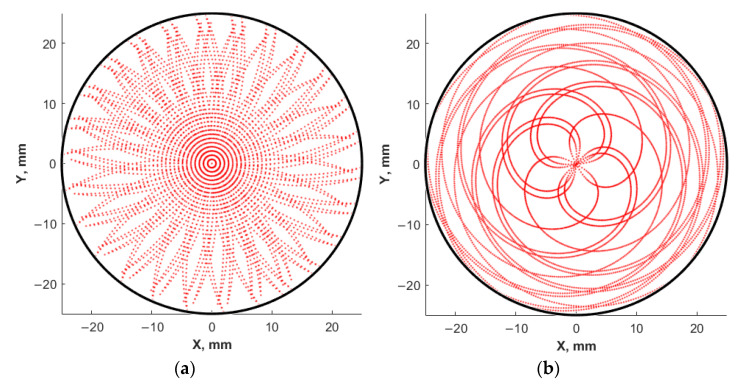
Trajectories of the laser beam movement along the target surface: scanning mode by the target with a stationary laser beam (**a**); scanning mode of the laser beam along the surface of a rotating target (**b**).

In the block for calculating of laser trajectory on target surface, the coordinates corresponding to each individual pulse are calculated, considering the operating modes of the scanning system and the parameters of the roll and rotation of the target. Figure 3 shows an example of the calculated trajectories of the laser beam along the target surface for various simulation parameters. Each point corresponds to one laser pulse, with the pulse repetition rate of 10 Hz, and the number of pulses is 6000.

The block for constructing the projection of the laser trajectory on the target surface onto the substrate is necessary to translate the array of coordinates of laser pulses from the reporting system associated with the center of the target to the reporting system associated with the center of the substrate, taking into account the parameters of the substrate rotation. It is necessary to calculate the distances *r* and *l* (Figure S1).

The film thickness calculation block specifies a set of points in a polar coordinate system. In the radial direction, the grid step is $dr=r_{2}-r_{1}=r_{3}-r_{2}=\ldots =r_{n}-r_{n-1}$, and in the corner $d\varphi =\varphi_{2}-\varphi_{1}=\varphi_{3}-\varphi_{2}=\ldots =\varphi_{m}-\varphi_{m-1}$. The resulting set of points can be represented as a matrix:

(7) $$\left[\begin{array}{cccccc} 0 & r_{1} & r_{2} & \ldots & r_{n-1} & r_{n}\\ \varphi_{1} & d_{1,1} & d_{1,2} & \ldots & d_{1,n-1} & d_{1,n}\\ \varphi_{2} & d_{2,1} & d_{2,2} & \ldots & d_{2,n-1} & d_{2,n}\\ \ldots & \ldots & \ldots & \ldots & \ldots & \ldots \\ \varphi_{m-1} & d_{m-1,1} & d_{m-1,2} & d_{m-1,3} & d_{m-1,n-1} & d_{m-1,n}\\ \varphi_{m} & d_{m,1} & d_{m,2} & d_{m,3} & d_{m,n-1} & d_{m,n} \end{array}\right]$$

where $d_{mn}$—thickness value at point ($r_{n},\varphi_{m}$).

Then Equation (3) for determining the film thickness at each point with coordinates ($r_{n},\varphi_{m}$) on the substrate surface has the form

(8) $$d_{m,n}=\frac{M_{e}}{\pi \rho_{u}h^{2}}\sum_{i=1}^{m}\sum_{j=1}^{n}{\left[1+{\left(l_{i,j}/h\right)}^{2}\right]}^{-2}.$$

According to the Equation (8), the film thickness *d* is determined, and the obtained value is written into the corresponding cell of the matrix. After receiving an array of thicknesses, the search for the most considerable value is performed. Using the maximum film thickness $d_{\max}$, one can calculate the relative thickness $d=d_{n}/d_{\max}$, which allows comparing the simulation results obtained at different values of technological parameters.

In the output modeling results block, the results are saved in text and graphic formats.

Based on the described methodology, we studied the influence of the scanning system modes on the film thickness distribution profile over a substrate with a diameter of 100 mm and 300 mm.

Experimental studies were carried to prove the proposed methodology. Nanocrystalline LiNbO_3_ films were obtained on a silicon substrate with a diameter of 100 mm using the Pioneer 180 PLD module of the NANOFAB NTK-9 cluster nanotechnological complex. An excimer KrF laser (λ = 248 nm) was used to ablate a LiNbO_3_ target with a purity of 99.9% (Kurt J. Lesker Company Ltd., East Sussex, UK). Energy density on the target surface is maintained at 1.5 J/cm^2^. The target-substrate distance (75 mm), number of pulses (36,000), pulse repetition rate (10 Hz), and laser pulses energy on the target surface (150 mJ) are kept constant. The morphology of the obtained films was studied by atomic force microscopy (AFM) using the Ntegra Probe Nano Laboratory (NT-MDT, Zelenograd, Russia). The experimental data were processed using the Image Analysis 3.5 software (NT-MDT, Zelenograd, Russia).

The study of the thickness of the LiNbO_3_ films was carried out by measuring the LiNbO_3_/Si structure height obtained by plasma-chemical etching. For this purpose, an FP-383 photoresist was applied to the surface of the LiNbO_3_ films by spinning at a rotation speed of 3000 rpm. After that, the photoresist film was pre-cured in the air for 10 min in order to prevent defect formation. Then, the sample was cured in an oven at 90 °C for 30 min. The sample was exposed to the UV radiation for 2 min through a photomask and then developed in a 5% aqueous solution of KOH, and hardbacked at 110 °C for 25 min on a hotplate. The obtained structure was processed in a module for plasma-chemical etching in a combined plasma of capacitive and inductive STE ICPe68 (SemiTEq St. Petersburg, Russia). Etching took place at a pressure of 2 Pa, a capacitive plasma source power of 35 W, an inductively coupled plasma source power of 400 W, a bias voltage of 75 V, a fluorinated SF_6_ gas flow of 15 sccm, and the etching time was 1 min. Photoresist residues were removed in dimethylformamide. In total, ten samples were used to fabricate structures in LiNbO_3_ films, the height of which was investigated by the Ntegra Laboratory in the semicontact AFM mode using NSG11 cantilevers.

## 3. Results and Discussion

**Figure 4:**
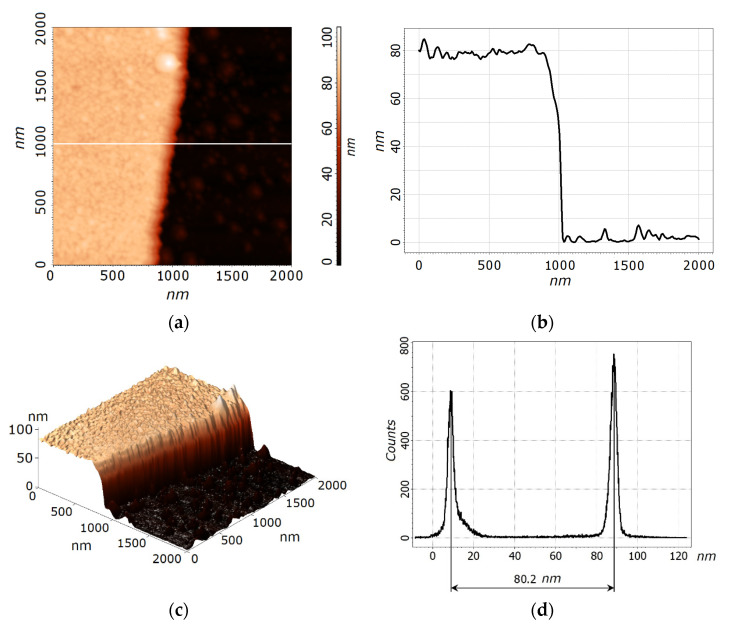
Experimental studies of a structure in a LiNbO_3_ film: AFM image (**a**); AFM cross-section (**b**) along the “white” line indicated in (**a**); 3D AFM reconstruction image (**c**); height histogram (**d**).

**Figure 5:**
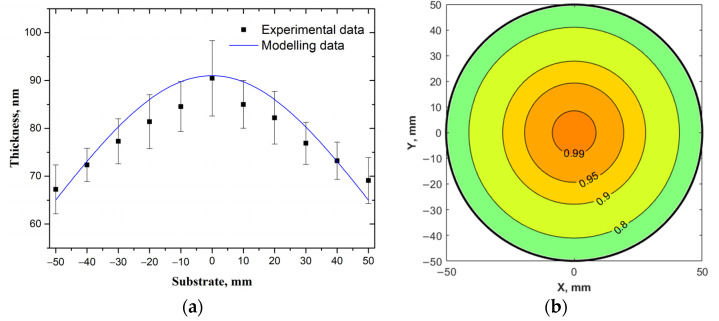
Comparison of the experimental results (points) and simulation (line) (**a**), as well as the distribution of the relative thickness of the LiNbO_3_ film on a substrate with a diameter of 100 mm (**b**).

Figure 4 shows the results of AFM studies of LiNbO_3_ film. Based on the obtained experimental results, the dependence of the distribution of the thickness of the LiNbO_3_ film over the surface of the silicon substrate is plotted (Figure 5).

To determine the LiNbO_3_ films thickness we used the approach described in detail in [47].

The obtained experimental results are in good agreement with the calculations based on the proposed Equations (1)–(8) for a substrate with a diameter of 100 mm. The thickness of the obtained LiNbO_3_ films increases from (67.2 ± 5.1) nm, reaching a maximum ((90.4 ± 7.9) nm) in the center of the substrate. The calculated thickness of the films varies from 63.6 nm to 89.1 nm, respectively. The change in the relative film thickness is less than 37% for the experiment and ~29% for the simulation results. The deviation of the simulation results from the experimental data does not exceed 8%. The parameter $\Omega_{95}$ is ~15%. The region with the thickness difference is 10% of the maximum, accounts for ~31% of the substrate surface. The discrepancy between the simulation and experiment may be associated with an uneven erosion of the target surface over multiple deposition cycles and a tilt of an ablation plume, as it is described in [23]. Thus, experimental studies confirm that the proposed methodology allows to calculate the film thickness’s value obtained by the PLD method.

**Figure 6:**
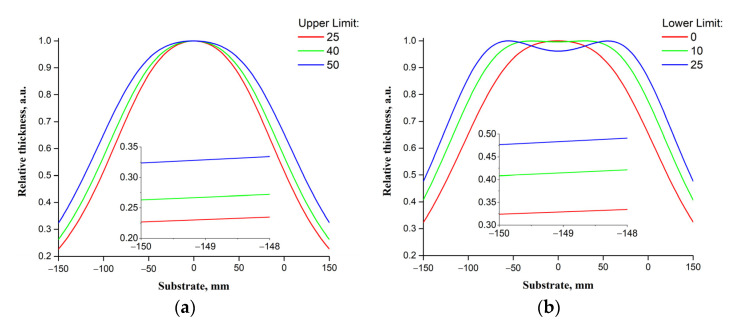
Dependencies of the relative thickness of the LiNbO_3_ film on the *Upper Limit* (**a**) and *Lower Limit* (**b**) parameters.

**Figure 7:**
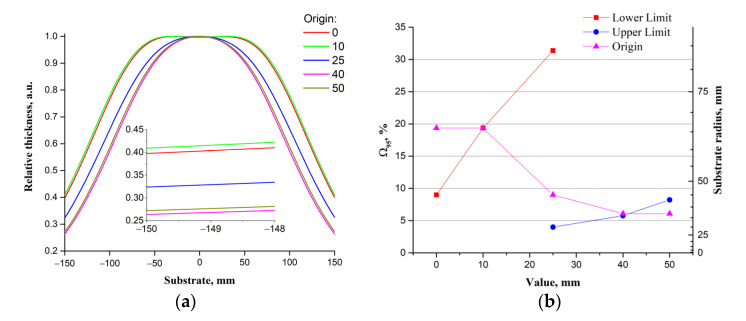
Dependencies of the relative film thickness on the *Origin* parameter (**a**) and $\Omega_{95}$ and substrate radius on the *Lower Limit*, *Upper Limit,* and *Origin* (**b**).

**Figure 8:**
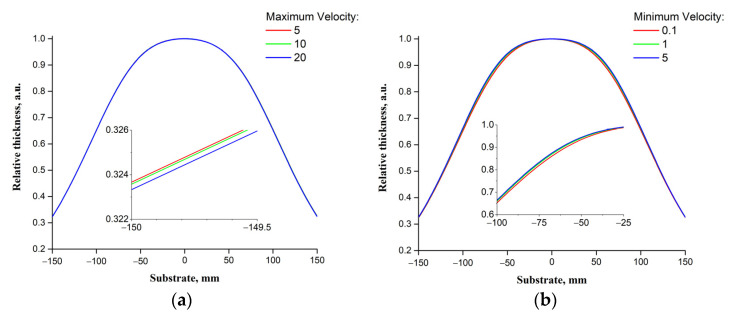
Dependencies of the relative thicknesses of the films on the parameters *Max Velocity* (**a**) and *Min Velocity* (**b**).

**Figure 9:**
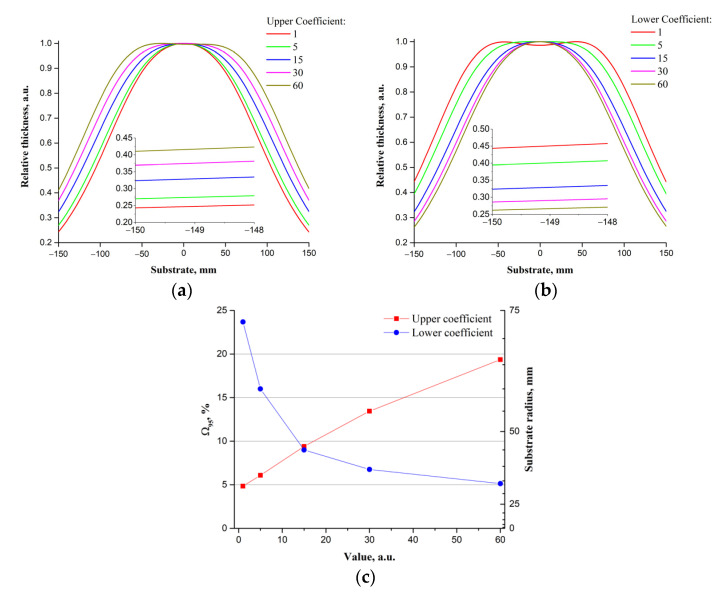
Dependencies of the relative film thicknesses on the parameters *Upper Coefficient* (**a**) and *Lower Coefficient* (**b**); $\Omega_{95}$ and substrate radius dependencies on the parameters *Upper Coefficient* and *Lower Coefficient* (**c**).

Figure 6 shows the relative thickness of the LiNbO_3_ films on the *Upper Limit* and *Lower Limit* parameters, calculated based on the developed Equations (1)–(8). Figure 6 shows the results of the study of the influence of scanning parameters on the uniformity of the thickness of the ZnO films. Insets in Figure 6, Figure 7, Figure 8 and Figure 9 provide a comparison of relative thicknesses for different cases at a higher scale. The appearance and change in the size of the “plateau” in the upper part of the dependence illustrate the physical meaning of the $\Omega_{95}$ parameter.

As the *Lower Limit* increases with the *Upper Limit* unchanged, the $\Omega_{95}$ parameter value increases from 9% to ~31%, while when the *Upper Limit* decreases with *the Lower Limit* unchanged, it decreases from ~8% to 4%. It can be associate with the fact that in the first case, we move the projection of the area of scanning of the target surface with a laser beam from the center of the substrate to the edge, and in the second, we bring it closer (Figure S1).

The results of studying the effect of the *Origin* parameter (Figure 7a) on the relative thickness of the films showed when the value of *Origin* decreases from 50 to 0, the value of $\Omega_{95}$ the parameter increases from 6% to ~20%. This effect is linked to the distance of the projection of the target area with laser beam moves at a speed of *Min Velocity* from the center of the substrate to the edge (Figure S1). Thus, the *Origin* parameter affects the relative film thickness by moving the projections of the regions with the maximum and minimum scanning speeds of the target surface (parameters *Min Velocity* and *Max Velocity*) relative to the center of the substrate. Figure 7b shows the influence of *Lower Limit*, *Upper Limit*, and *Origin* on $\Omega_{95}$ parameter and substrate radius.

A change in the maximum and minimum speeds of the laser beam movement over the target surface has practically no effect on the distribution of the relative thickness of the resulting films (Figure 8). Since the *Min Velocity* and *Max Velocity* parameters do not affect the projections of areas with high and low scanning speeds of the target surface relative to the center of the substrate, but only determines the scanning speed.

Unlike *Max Velocity* and *Min Velocity*, the *Upper Coefficient* and *Lower Coefficient* parameters have a more significant effect on the distribution of the relative thickness of the films (Figure 8). It is linked to the possibility of influencing the smoothness of the transition from *Min Velocity* to *Max Velocity* in the (*Lower Limit*, *Origin*) and *Max Velocity* in *Min Velocity* on the (*Origin*, *Upper Limit*) segment and, as a consequence, change the size of the areas that have the maximum and minimum scanning speeds. Therefore, with the values of *Upper Coefficient* and *Lower Coefficient* close to unity, the scanning speed practically does not change when switching from *Max Velocity* to *Min Velocity*, while at values above 60, the speed changes abruptly. Thus, increasing the *Upper Coefficient* value in the (*Origin*, *Upper Limit*) segment increases the area with the scanning speed is equal to the *Min Velocity*, which leads to the displacement of the projection of the target area with the minimum scanning speed from the center to the edge of the substrate. Figure 9c shows the $\Omega_{95}$ and substrate diameter dependencies on the value of the parameters *Upper Coefficient* and *Lower Coefficient*. As the *Upper Coefficient* parameter increases with a constant *Lower Coefficient*, $\Omega_{95}$ increases from ~5% to ~20%, and with an increase in the *Lower Coefficient* parameter, with an unchanged *Upper Coefficient* $\Omega_{95}$ decreases from ~24% to ~5%.

Based on the results of the performed calculations, recommendations are proposed that allow increasing the thickness uniformity of the films formed by the PLD method.

It has been established that increasing the operating mode of the scanning system with the projections of the target area with a low scanning speed will be shifted from the center to the edge of the substrate. This effect can be achieved in several ways:

– changing the scanning range—*Upper Limit* and *Lower Limit* parameters;
– changing the coordinate in which the scanning speed reaches its maximum—*Origin* parameter;
– varying the smoothness of changes in scanning speeds—*Upper Coefficient* и *Lower Coefficient* parameters.

It should be noted that the first method should be used to a limited extent since the *Upper Limit* and *Lower Limit* parameters strongly affect the uniformity of the target erosion.

**Figure 10:**
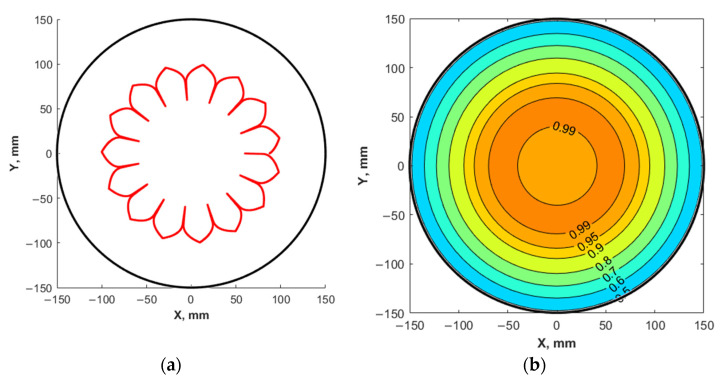
Projection of the trajectory of the laser beam along the target surface onto the substrate surface (**a**) and the distribution of the relative thickness (**b**) of the obtained film on a substrate with a diameter of 300 mm.

Using the calculation results, the operating modes of the scanning system with a high $\Omega_{95}$ value when films are deposited on substrates with a diameter of 300 mm were determined: *Upper Limit =* 50, *Lower Limit* = 10, *Origin* = 30, *Max Velocity* = 5, *Min Velocity =* 0.1, *Upper Coefficient* = 60, and *Lower Coefficient* = 1. For these parameters, the projection onto the surface of the substrate of the laser beam trajectory along the target surface and the distribution of the relative thickness of the LiNbO_3_ film on the substrate were calculated (Figure 10).

The projection of the laser beam trajectory onto the surface of the substrate (Figure 10a) makes it possible to estimate the location where ablation proceeds relative to the center of the substrate. Figure 10b shows the spatial distribution of the relative thickness of the obtained film over the surface of a substrate with a diameter of 300 mm. It was found that the change in the relative thickness of the film does not exceed 50%, but $\Omega_{95}$ is ~31%, while the region with the difference in thickness is 10%, of which the maximum is ~38%.

## 4. Conclusions

The methodology development results allow calculating the film thickness distribution profile over the surface of the substrate, taking into account the design and technical parameters of the PLD equipment. The film thickness distribution profile on a substrate with a diameter of 100 mm is calculated considering the design features and operating values of the technological parameters of the Pioneer 180 PLD module of cluster nanotechnological complex NANOFAB NTK-9. The thickness of the obtained LiNbO_3_ films increases from (67.2 ± 5.1) nm, reaching a maximum ((90.4 ± 7.8) nm) in the center of the substrate. The calculated thickness of the films varies from 63.6 nm to 89.1 nm, respectively. The change in the relative film thickness is less than 37% for the experiment and ~ 29% for the simulation results. A good correlation of the results of theoretical calculations with experimental data is shown: the discrepancy between the model and experiment does not exceed 8%. Moreover, the developed methodology allows to solve the inverse task and scale it, overcoming relevant issues in modern micro- and nanoelectronic technology. It is possible to determine the technological parameters of the PLD module for obtaining a film with a controlled uniformity of thickness distribution using the modeling results. The analysis of the influence of the operating parameters of the scanning system (Table 1) on the uniformity of obtaining films on a substrate with a diameter of 300 mm is carried out. Recommendations for obtain films with controlled irregularity by PLD method are proposed: (*Upper Limit = 50*, *Lower Limit* = 10, *Origin* = 30, *Max Velocity* = 5, *Min Velocity =* 0.1, *Upper Coefficient* = 60, and *Lower Coefficient* = 1). Based on obtained results, the operating modes of the scanning system are proposed, which allows obtaining films with a relative thickness difference not exceeding 50%. In this case, the region in which the difference in thickness is less than 5% ($\Omega_{95}$) accounts for 31% of the substrate surface, and the region in which the difference in thickness is 10% of the maximum is ~38%.

The obtained results can be used to expand the capabilities of the large-area PLD technology, which will speed up combining the technology of multicomponent oxide films laser deposition with the silicon technology of micro- and nanoelectronics and molecular device electronic to create MEMS and new generation of energy conversion devices and sensors. Moreover, the developed methodology might be modified and scaled to calculate the thickness profile of different materials films obtained using various PLD equipment (Solmates B.V., PVD Products Inc, and Neocera LCC).

## Figures and Tables

**Table 1:** Modeling parameters.

**Technological Parameters**					
Number of laser pulses	36,000				
Laser pulse repetition rate	10 Hz				
Substrate diameter	300 mm				
Target diameter	50 mm				
Target-substrate distance	75 mm				
Target rotation speed	10 °/s				
Substrate rotation speed	0.1 °/s				
**Scanning System Parameters (default parameters are highlighted)**					
*Origin*	0	10	25	40	50
*Upper Limit*	25		40	50	
*Lower Limit*	0		10	25	
*Maximum Velocity*	5		10	20	
*Minimum Velocity*	0.1		1	5	
*Lower Coefficient*	1	5	15	30	60
*Upper Coefficient*					

## Data Availability

Data sharing is not applicable to this article.

## Supplementary Materials

The following are available online at https://www.mdpi.com/article/10.3390/ma14174854/s1, Figure S1: Target material evaporation from a cell $dA_{e}$ by a laser beam on substrate surface element $dA_{r}$, (a) and Pioneer 180 PLD software (b), Figure S2: Example of practical use of the obtained dependencies, Video S1: Scanning mode by the target with a stationary laser beam, Video S2: Scanning mode of the laser beam along the surface of a rotating target.
