# Supplementary material for: Towards Scalable Large-Area Pulsed Laser Deposition
Source: Materials (Basel). 2021 Aug 26;14(17):4854. doi: 10.3390/ma14174854 (PMC8432691; doi:10.3390/ma14174854)
Supplement: Supplementary file 1 [file materials-14-04854-s001.zip › materials-1330824-supporting.pdf]

materials

MDPI

Article

Towards Scalable Large-Area Pulsed Laser Deposition

Zakhar Vakulov [1,*,†], Daniil Khakhulin [2,†], Evgeny Zamburg [3], Alexander Mikhaylichenko [4], Vladimir A. Smirnov [5], Roman Tominov [5], Viktor S. Klimin [5] and Oleg A. Ageev [5,6]

[1] Federal Research Centre The Southern Scientific Centre of the Russian Academy of Sciences (SSC RAS), 41 Chekhov St., 344006 Rostov-on-Don, Russia
[2] Research Laboratory of Functional Nanomaterials Technology, Southern Federal University, 2 Shevchenko St., 347922 Taganrog, Russia; dhahulin@sfedu.ru
[3] Department of Electrical & Computer Engineering, National University of Singapore, 4 Engineering Drive 3, Singapore 117583, Singapore; zamburg@nus.edu.sg
[4] FORS Development Center, 3, Trifonovskiy Tupik, 129272 Moscow, Russia; alexandrVM@bk.ru
[5] Institute of Nanotechnologies, Electronics and Equipment Engineering, Southern Federal University, 2 Shevchenko St., 347922 Taganrog, Russia; vasmirnov@sfedu.ru (V.A.S.), tominov@sfedu.ru (R.T.), kliminvs@sfedu.ru (V.S.K.); ageev@sfedu.ru (O.A.A.)
[6] Research and Education Centre 'Nanotechnologies', Southern Federal University, 2 Shevchenko St., 347922 Taganrog, Russia
* Correspondence: vakulov@ssc-ras.ru
† These authors contributed equally to this work.

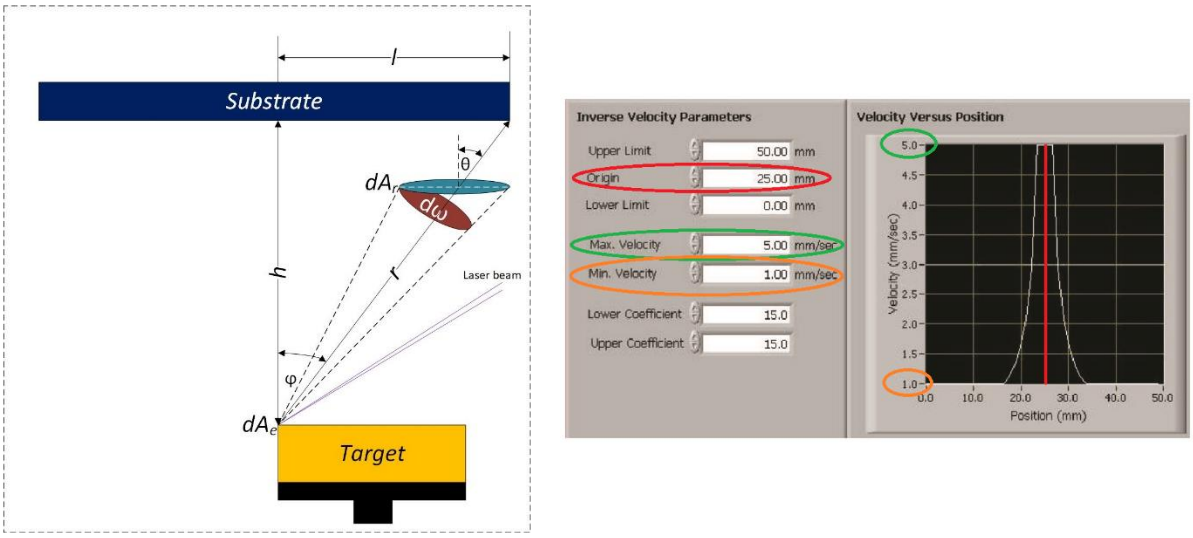

(**a**) (**b**)

Figure S1. Target material evaporation from a cell dA_e by a laser beam on substrate surface element dA_r (**a**) and Pioneer 180 PLD software (**b**).

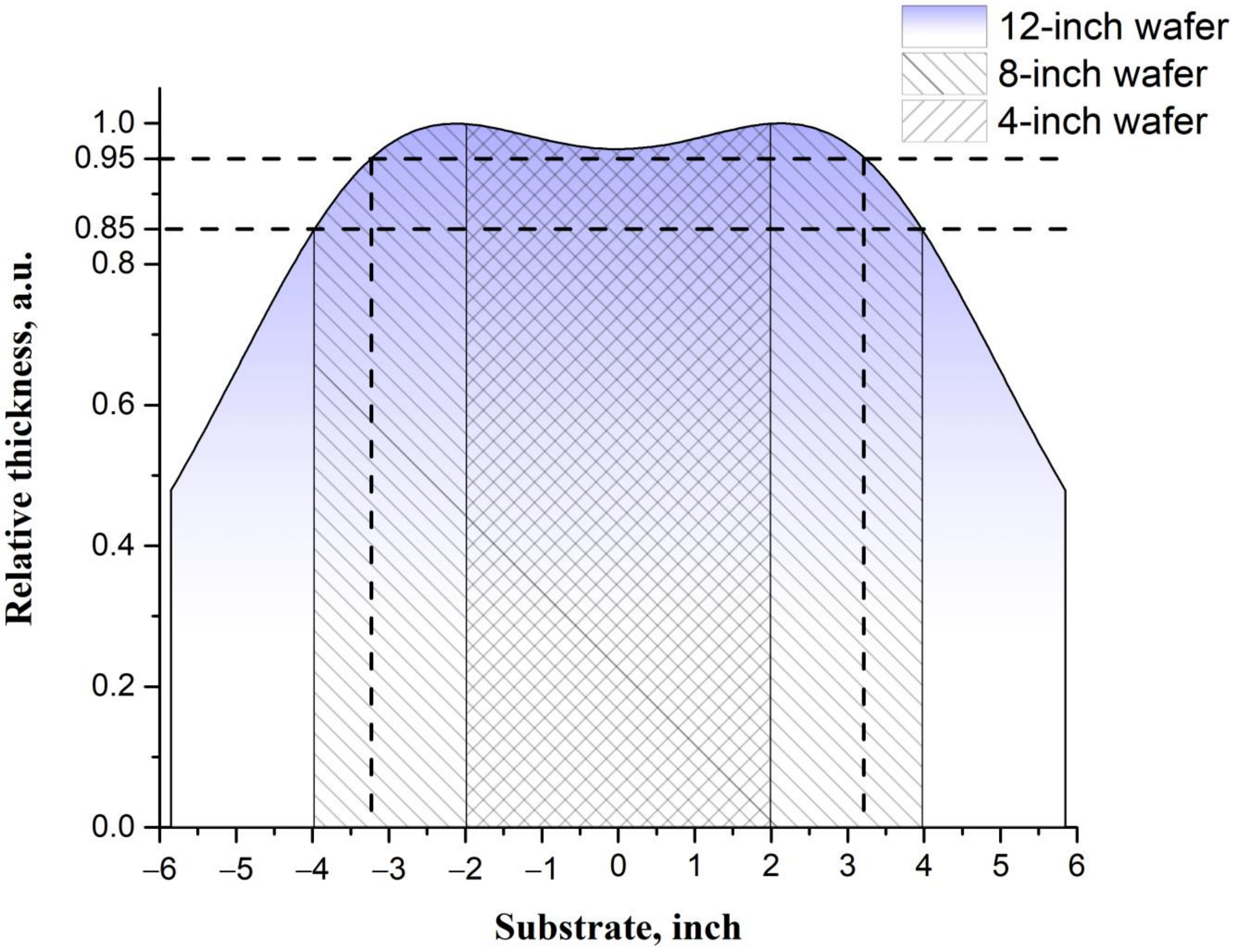

Figure S2. Example of practical use of the obtained dependencies.
